# Aversive conditioning increases short-term wariness but does not change habitat use in black bears associated with conflict

**DOI:** 10.1371/journal.pone.0295989

**Published:** 2024-01-02

**Authors:** Lori Homstol, Sage Raymond, Claire Edwards, Anthony N. Hamilton, Colleen Cassady St. Clair

**Affiliations:** 1 Department of Biological Sciences, University of Alberta, Edmonton, Alberta, Canada; 2 Provincial Government of British Columbia, Victoria, British Columbia, Canada; 3 Jasper National Park, Jasper, Alberta, Canada; Cheetah Conservation Fund, Namibia University of Science and Technology, NAMIBIA

## Abstract

Conflict between humans and black bears (*Ursus americanus*) occurs throughout North America with increasing public demand to replace lethal management with non-lethal methods, such as aversive conditioning (AC). AC aims to teach animals to associate negative stimuli with humans or their infrastructure. We sought to test the efficacy of AC using radio-collared black bears in Whistler, British Columbia, by monitoring individuals and assigning those in conflict with people to control or treatment groups. We measured wariness using overt reaction distance, displacement distance, and reaction to researchers before, during and after executing 3–5-day AC programs that consisted of launching projectiles at bears in the treatment group. We also assessed predictors of successful AC events (i.e., leaving at a run), changes in bear use of human-dominated habitat during the day and at night, and the effects of including a sound stimulus to signal the beginning and end of AC events. Among treated bears, overt reaction distance increased by 46.5% and displacement distance increased by 69.0% following AC programs, whereas both overt reaction distance and displacement distance decreased over time among control group bears. Each additional AC event during the previous 30 days increased likelihood of bear departure in response to researcher presence by 4.5%. The success of AC events varied among individuals, declined with distance to cover, and increased with exposure to previous AC events. Projectiles launched from guns were slightly more effective at causing bears to displace compared to those launched from slingshots, and sound stimuli decreased the likelihood of a successful AC event. AC did not alter diurnal use by bears of human-dominated habitat. Our results suggest that AC effectively increases short-term wariness in black bears but does not alter bear use of human-dominated spaces, highlighting the importance of proactive attractant management and prevention of food conditioning.

## Introduction

Global urbanization and human population growth have reduced populations of many wildlife species [1, 2], especially carnivores [3]. While most large carnivores are absent from urbanized areas, several species co-occur with people in human-dominated regions [4, 5], where they are often associated with human-wildlife conflict [6, 7]. The North American black bear (*Ursus americanus*) is a large carnivore that can adapt to human-dominated areas and is associated with increasing human-wildlife conflict [8, 9] throughout North America e.g., [6, 10, 11], including urban areas [12–14] and suburban ones [7, 9]. Human-black bear conflict occurs when bears access garbage [6, 15], damage property [12, 16], feed on pets or livestock [17], are involved in vehicle collisions [18], and, rarely, attack humans [8, 19]. Food conditioning considerably increases human-black bear conflict [20–22], as occurs with other urban-adapted animals [23, 24]. Conflict is also linked to habitat encroachment associated with human population growth [25] and the seasonal availability of natural foods in nearby habitat [14, 15].

Because black bears can pose a safety risk to humans [8, 19, 26], wildlife managers must address individuals associated with conflict. Public education is considered crucial for conflict reduction [9, 27, 28], but conflict often persists, requiring further management [29]. Two of the primary strategies for managing black bears associated with conflict (hereafter ‘conflict bears’) include lethal management and relocation [9, 30]. Lethal management can be effective in that it selectively removes conflict animals [31], but removals may also render areas suitable for entry by new bears that may also come into conflict with people [31, 32]. In urban areas, lethal control is often impractical and it is inappropriate where bears are protected, such as the Louisiana black bear (*U*. *americanus luteolus* in Louisiana, USA [33]) and the brown bear (*U*. *arctos*) in parts of Europe [34]. Additionally, lethal management is increasingly unpalatable to the general public [31, 35, 36], with many residents considering lethal responses to conflict black bears unacceptable [30]. Intolerance for lethal management appears to be especially prominent in urban areas [37] where conflict is more likely [4, 12, 38].

Relocation, defined as movement within home ranges [39], of conflict bears is often preferred by human inhabitants of cities [27], but it is resource-intensive and therefore impractical for ubiquitous species that are not threatened. Most (85%) wildlife agencies that reported using relocation to limit human-black bear conflict considered it to be an ineffective management tool [27]. If suitable habitat or anthropogenic attractants remain, new individuals will typically inhabit the area previously occupied by the relocated animal [31, 32], and bears may travel long distances to return to their former home range [40, 41]. Survival of bears that are translocated, defined as movement beyond their home ranges [32, 39], is often low, perhaps owing to competition with established resident bears or the inability to adapt to a new location [31, 32], which could be related to learned conflict behaviour [42] or memory of landscape resources and phenology [43]. The limitations of lethal management and relocation, especially in urban contexts, promote the need for effective, non-lethal alternatives that reduce human-black bear conflict.

Aversive conditioning (AC) is a non-lethal tool that wildlife managers increasingly employ when addressing conflict wildlife, including bears [44]. AC is a process in which managers administer negative physical or psychological stimuli to promote a negative association with people, human infrastructure, and anthropogenic food sources [31, 45], ultimately reducing animal tolerance towards humans, with the aim of decreasing human-wildlife conflict [46]. AC techniques applied to bears have included loud noises [27, 34, 47], non-lethal projectiles [34, 45, 48], pyrotechnics [34], and chasing bears, with or without dogs [33, 48, 49]. AC differs from the similar practice of hazing, which typically involves deterring a bear from an immediate conflict situation with the same methods but without follow-up action promoting learned avoidance of people. Compared to other management options, the public generally accepts AC of black bears, especially when bears are one-time offenders [30], and AC is employed by 64% of surveyed management agencies [27].

Despite public acceptance and frequent use by managing agencies, AC has been variably successful at reducing human-bear conflict [9, 29, 45]. Several studies suggest AC is effective at reducing conflict behaviour and increasing wariness in the short-term, in both black bears [33, 48, 50, 51] and grizzly bears [47, 52]. Although bears typically flee from launched projectiles, some bears soon return [45, 49, 52] or displace short distances and remain in residential areas [38]. Food conditioned bears are less likely to respond well to AC; an AC program in Sequoia National Park, USA, successfully prevented naïve black bears from becoming food-conditioned, but it failed to cause behavioural changes in 41% of bears that were already food-conditioned [48]. Similarly, black bears subjected to an AC program in Juneau, USA, persistently engaged in conflict behaviour, which may have resulted from the continued presence of accessible garbage [38], which decreases AC program effectiveness [52]. Additionally, AC appears to be most effective in early stages of habituation or food conditioning [45]. Age, sex and health of bears may also affect the outcome of AC. Compared to healthy adult bears, yearling black bears [48] and diseased grizzly bears [52] were less likely to modify their behaviour in response to AC, but individuals may respond differently, even when age-sex class is controlled [48–50, 52].

For AC to be effective, bears must associate the unconditioned stimulus (i.e., the painful stimulus) with a conditioning stimulus, which is an environmental condition that is occurring when the unconditioned stimulus is applied [53]. Most studies rely on elements of the conflict situation (e.g., location, food odours etc.) to represent the conditioning stimulus [38], but research on the neurological pathways of learning mechanisms in mammals suggests that auditory conditioning stimuli may be more effective [53]. This idea was explicitly tested in an AC study of female grizzly bears, in which researchers used calls of non-native birds as a conditioning stimulus, but a small sample size led to inconclusive results [52]. In another AC study, researchers noticed black bears responding to the auditory cue of a gun being cocked, providing anecdotal support for a learned association between an auditory cue and a painful stimulus [38]. AC programs appear to be more effective when bears are subjected to AC while they engage in conflict behaviour e.g., [48, 50, 52] as opposed to programs that primarily feature hazing following the release of a captured bear; so-called hard release [33, 34, 49]. This difference would be expected perhaps because of the spatial or temporal separation between the conditioning and unconditioned stimuli e.g., [54]. Even when bears are treated as they engage in conflict behaviour, repeated treatments are often required for bears to generalize learning among conflict sites [52]. The various uncertainties about the effectiveness of AC, in combination with its ubiquity and acceptance as a management technique, emphasize the need to better understand where, when, and how AC should be conducted to manage conflict bears.

In this study, our goal was to test the effectiveness of AC for changing the behaviour of conflict black bears while addressing three main objectives. First, we determined whether AC increases bear wariness, which we assessed using overt reaction distance, displacement distance, and reaction to researchers. Second, we determined the predictors of successful AC events, defined as the displacement of a bear to cover (i.e., vegetation that prevented people from seeing a bear), and explicitly compared bear responses to marbles launched with a slingshot to bean bags, cracker shells, or rubber slugs launched from a shotgun or paintball gun. For some bears, we included a sound conditioning stimulus as part of the AC program. Third, we determined how AC affected bear use of human-dominated habitat (i.e., paved roads and residential communities) during the day and at night. By achieving these objectives, we hoped to provide information that could be integrated into evidence-based protocols for bear management.

## Materials and methods

### Study area

The study occurred in the Resort Municipality of Whistler, British Columbia (BC), in the Coast Mountains of southwestern British Columbia, primarily in the Coastal Western Hemlock biogeoclimatic zone [55]. Capture and collaring of black bears began in 2005, and AC programs occurred between 2007 and 2008. At the time of the study, Whistler had an annual human population of ~10000 permanent residents and ~1.8 million tourists [56], and a black bear population of ~ 100 bears, with a density of ~1 bear/ km^2^ [57]. Bear populations were supported by abundant natural and anthropogenic food and extensive forested areas within city limits [57]; because attractants were present throughout the active season (i.e., April through November), bears accessed attractants and sometimes caused conflict throughout this period. The study therefore occurred throughout the active season. Shrub species important for bears include huckleberry (*Vaccinium membranaceum*) and blueberry (*V*. *ovalifolium and V*. *alaskensis*), highbush cranberry (*Viburnum trilobum*), Saskatoon berry (*Amelanchier alnifolia*) and sitka mountain ash (*Sorbus sitchensis*) [55].

### Field methods

We collaborated with the British Columbia Conservation Officer Service to capture bears using culvert traps and free-range darting. We immobilized bears by injecting a combination of Tiletamine and Zolazepam (Telazol) intramuscularly (100–300 mg/mL) [58]. While immobilized, we sexed bears, recorded body measurements, and fitted individuals with a Lotek 4400S model GPS or Telonics VHF radio-collar (S1 Table). To prevent interactions with humans or other bears, researchers monitored the area while animals recovered at or near capture locations [38].

To assess candidate bears for designation as being in conflict and inclusion in the AC program, we monitored the locations of collared bears every few days throughout months when bears were active (April—November) in the study period (2007–2008) using a four-element Yagi antenna mounted on a vehicle (radio-collared bears) or by examining GPS data generated at hourly fixes (GPS-collared bears). We used location information to find bears and observe them opportunistically, with variation owing to technician availability and bear location. Upon detecting bears, we noted their behaviour and any evidence of recent access to anthropogenic attractants (e.g., feeding on mowed grass, accessing or investigating garbage, domestic fruit trees, or birdseed). Based on at least five direct observations, we classified individuals as conflict bears if we regularly observed them engaging in conflict with humans, either by accessing or attempting to access anthropogenic foods (above), by demonstrating a lack of wariness to humans in urban areas, or frequent use of human-dominated areas such as parks, golf courses, and residential neighbourhoods, especially during the day. These observations were opportunistic and depended on bear location and technician availability; typically, they occurred within one week. Some conflict bears were likely food-conditioned, while others were likely habituated to people but not dependent on anthropogenic subsidies [59]. We randomly assigned all conflict individuals to either a Control Group or an AC Group, at a ratio of approximately 3 individuals in the AC Group per individual in the Control Group [33]. If bears in the Control Group exhibited increasing conflict behaviour, we re-assigned them to the AC Group to promote public safety [52]. Among individuals in the AC Group, we randomly assigned individuals to be treated with or without sound.

Before beginning AC programs, we determined baseline wariness for all individuals in the study. We approached bears at a walk until we were up to 10-m away, and we determined Overt Reaction Distance, defined as the distance at which the bear visibly demonstrated that it had noticed us [26]. This behaviour was typified by bears stopping the activity they were previously engaged in and focusing on the approaching researcher. We also recorded displacement distance, defined as the distance between the researcher and the bear when the bear departed [26], which other researchers refer to as flight response distance [60] or flight initiation distance [61]. Because the initial distance between the animal and an approaching entity can affect displacement distance [62], we also recorded start distance. We generally used a range finder to quantify distances, but we occasionally estimated these values when direct measurement was not possible. Because various factors can affect overt reaction and displacement distance [26], we recorded these measurements only when bears were not actively accessing or attempting to access anthropogenic attractants, were not within 50 m of humans or other bears, and were between 1 and 50 meters from cover, which we defined as vegetation that prevented people from detecting it visually. We used between 1 and 5 approaches, which typically occurred within 1 week, to determine baseline responses before conditioning ($\bar{\text{x}}=2.5\pm 1.1$ SD).

After acquiring baseline data, we began AC programs for bears in the AC Group, which we developed according to methods pioneered by Hunt [63]. We began AC programs between 24 hours and one week after the end of the final wariness measurement with exact timing depending on technician availability. AC programs ran for three to five days during daylight hours, during which researchers followed the target bear closely [33] and conducted AC each time the bear was observed engaging in the conflict behaviours above [38], usually access or attempted access to anthropogenic attractants or use of human-dominated spaces during daylight hours. While AC was primarily conducted during daylight hours, we continued to follow and treat bears after dusk and into the night when we observed individuals using human-dominated spaces after dark. Our primary AC tactic was to launch projectiles when bears were 15 – 35m away. When conservation officers were available, projectiles of rubber bullets, bean bags, or cracker shells were launched from pump action 12-gauge shotguns or paintballs from paintball guns [34]. When conservation offices were unavailable, researchers used slingshots to launch marbles at bears that were < 20m away [48]. For bears in the AC Group treated with sound, we blew a whistle before launching projectiles and rang a bell to signal the end of the AC event when the bear had entered cover and was no longer visible. We recorded bear response to researchers prior to beginning AC and bear response to AC (i.e., launching projectiles) as leaving at a run, leaving at a walk, or failing to displace. We opportunistically recorded the bear’s distance to cover, and start distance, overt reaction distance, and displacement distance. To assess ongoing wariness in Control Group bears, we continued to approach these individuals without launching projectiles. All field methods were approved by the University of Alberta animal welfare committee (Protocol 542905).

### Statistical methods

To determine the impacts of AC programs on overt reaction and displacement distance, we calculated mean overt reaction and displacement distances for each individual. For individuals in the AC Group, we developed pre and post-treatment values by averaging measurements collected during the week prior to the beginning of the AC program and the week following the end of the AC program, respectively. For individuals in the Control Group, we used a random number generator to arbitrarily select a three-to-five-day period (comparable to treated bears), and we calculated mean overt reaction distance and displacement distance during the week prior to and following this period. To better accommodate the relationship between the number of variables and the limited sample size, we calculated the percent change in overt reaction distance and displacement distance for each individual. We then used linear regression to predict the percent change in overt reaction distance or displacement distance, testing the influence of treatment type (i.e., Control or AC Group), and sex. To ensure there was no confound between start distance and pre and post-treatment, we used t-tests to compare average start distance for pre and post-treatment periods for both the Control and AC Groups. We used a similar modelling approach to test the influence of sound treatment (i.e., Sound or No Sound) on percent change in overt reaction distance and displacement distance among bears in the AC Group. We compared models containing different sets of predictors using Akaike Information Criterion adjusted for small sample sizes (AIC_c_), compared models to null models using likelihood ratio tests [64, 65] and assessed model performance using adjusted R^2^ [64].

To further assess changes in bear wariness, we considered bear response to researchers. For this analysis, we considered only bears subjected to AC. We coded bear response to researchers as 1 if the bear left at a run or a walk before the researchers began conducting AC and launching projectiles and as 0 if the bear did not displace upon researcher arrival. Because of the importance of individual variation in the success of AC programs [48, 52, 66], we tested the influence of individual ID using a Fisher Test [67] and used generalised linear mixed effects models (GLMMs) with bear ID as a random effect. We used logistic regression (binomial distribution, logit link) to test the effects of sex, distance to cover (m), start distance (m), whether a whistle was blown, and the previous number of AC events experienced by a bear in the past 30 days. To determine the broad importance of each variable, we initially developed univariate models, which we compared to null models using AIC_c_ and likelihood ratio tests [64, 65]. We then developed a model using the most predictive variable (i.e., the variable that resulted in the lowest *P*-value when compared to the null model using a likelihood ratio test) and iteratively added variables that were associated with 95% confidence intervals that did not overlap 0 (univariate). We retained variables when they improved model performance according to AIC_c_ and likelihood ratio tests. This modelling approach allowed us to use as much of the dataset as possible, given missing data. We evaluated model performance using Nakagawa’s pseudo R^2^ for mixed effects models [68].

To determine the predictors of successful individual AC events, we considered all AC events for which bear reaction was recorded. Because we expected bears to show heightened responses to AC compared to researcher presence, we classified the event as successful (1) if the bear left at a run and as unsuccessful (0) if the bear left at a walk or failed to displace. We used logistic regression (binomial distribution, logit link), included bear ID as a random effect, and tested the influence of the number of AC events in the previous 30 days, sex, distance to cover (m), start distance (m), whether a whistle was blown, and projectile source (gun vs. slingshot) as fixed effects. We tested each variable individually to maximize statistical power, while retaining bear ID as a random effect, to identify candidate variables for subsequent, multivariate models [65]. We retained only the variables that lowered AIC_c_ and improved model performance compared to the null model according to likelihood ratio tests [64, 65], and we combined these terms in a final explanatory model, which we evaluated using Nakagawa pseudo R^2^ for mixed effect models [68].

To study spatial and temporal responses of bears to AC, we considered only bears fitted with GPS collars that recorded locations at hourly intervals. We classified location fixes as occurring during the day (i.e., between sunrise and sunset) or at night. We developed a polygon representing human-dominated habitat in Whistler, which included all paved roads and residential communities. For each GPS location, we calculated Euclidean distance to the human-dominated habitat polygon, and the proportion of a 100-m radius circular buffer occupied by human-dominated habitat. For both distance to human-dominated habitat and proportion of a 100-m radius buffer occupied by human-dominated habitat, we developed GLMMs that included bear ID as a random effect and an interaction between Time (i.e., pre or post-treatment) and Treatment Type (i.e., AC or Control Group) as a fixed effect. For bears in the AC Group, we defined the pre and post-AC periods as the three days prior to and following the execution of the AC program. For individuals in the Control Group, we used a random number generator to arbitrarily select a start date for the pre-treatment period; we began the post-treatment period six days later. We completed all geographic analyses with ArcGIS 9.3, and we completed statistical analyses and developed figures using RStudio Version 4.0.3.

## Results

### Bear capture

Between 2005 and 2007, we captured 28 black bears (10 female, 18 male) that were later classified as conflict individuals and included in this study (S1 Table). We fitted nine bears (5 females and 4 males) with GPS collars and the remaining bears with VHF radio collars. Of the bears with radio collars, five females and 12 males were assigned to the AC Group, and one female and two males were assigned to the Control Group. Seven bears in the AC Group were assigned as individuals that would be treated with sound. Of the bears with GPS collars, two females and three males were included in the AC Group, and three females and one male were included in the Control Group. Two bears in the study (one radio-collared male and one GPS-collared female) were initially included in the Control Group but were later subjected to AC programs following observed escalation in conflict behaviours that others have associated with public safety [52].

### Bear wariness

**Fig 1:**
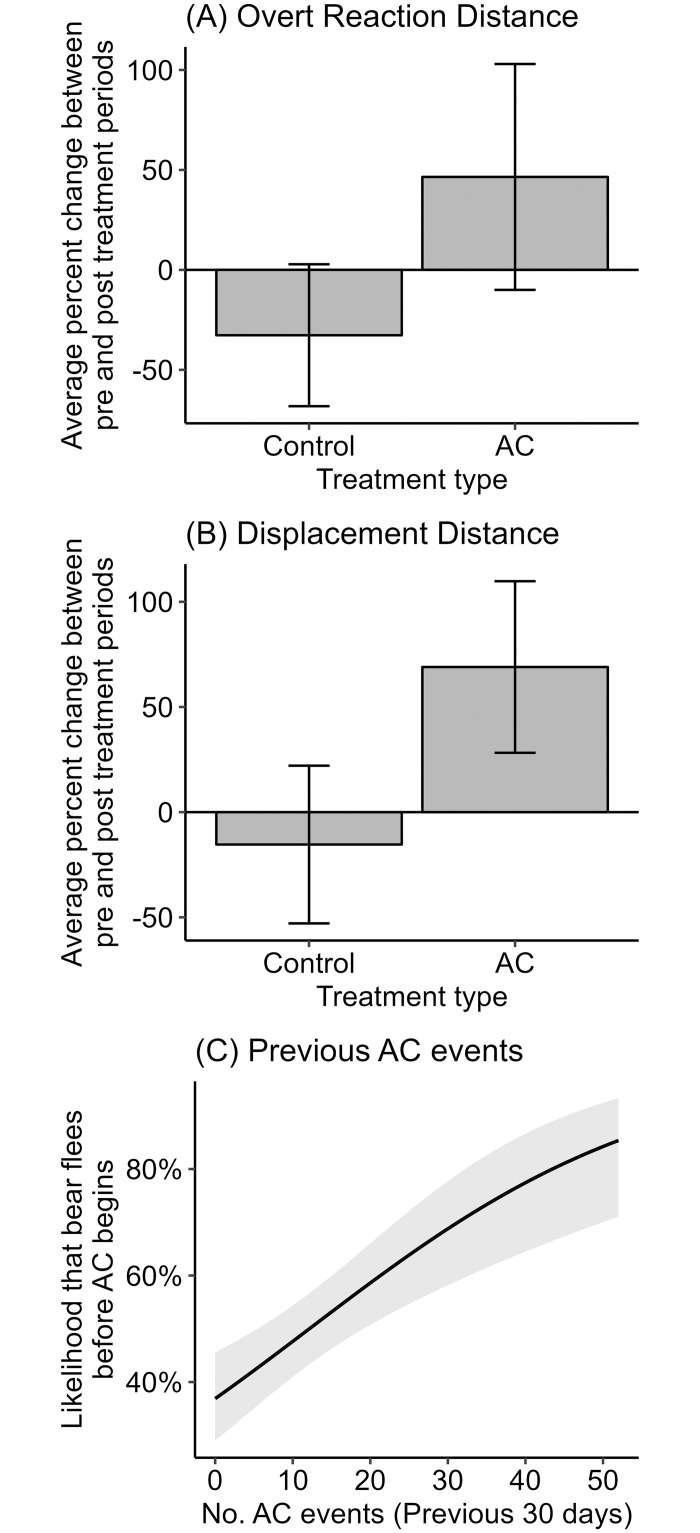
The effects of AC programs on three metrics of black bear wariness, Whistler BC, 2007–2008. A and B show the average observed percent change in overt reaction distance and displacement distance among bears in the AC Group and the Control Group. Error bars represent standard error. C shows the predicted effect of the number of AC events conducted during the previous 30 days on the likelihood that a bear will flee from researchers prior to their beginning AC treatment.

To assess the influence of AC programs on percent change in overt reaction distance and displacement distance, our sample (*n* = 22) included 11 individuals in each of 2007 and 2008, which included 10 females and 12 males. Of the bears in the AC Group (*n* = 14), seven individuals were subjected to sound treatments, and seven were not. Most bears were treated in only one year, but four individuals were subjected to AC programs in both 2007 and 2008, and we used individual bear-year combinations in our analysis. To assess the effects of pseudoreplication caused by reusing bears, we omitted data from a randomly selected year for each replicated individual, and repeated the analysis (*n* = 18), achieving results that were categorically the same. There was no difference in start distance between the pre and post periods in the Control Group (t = 1.43, df = 13.1, *P* = 0.176) or the AC Group (t = 0.382, df = 21.6, *P* = 0.7063), so we did not include this predictor in models. Average overt reaction distance calculated from data collected for bears in the AC Group increased 46.5% from 27.7m ± 19.8 (SD) to 32.3 m ± 10.6 (β = 79.2, CI = 32.8, 125.6, *P* = 0.002; Fig 1A), while bears in the Control Group exhibited an average decrease in overt reaction distance of 32.7% from 42.2m ± 30.0 to 24.0m ± 11.3 (Fig 1A). A model that included Treatment Group fit the data significantly better than the null model (ΔAIC_c_ = 8.1, *P* = 0.002). Adding sex to the model worsened model fit (ΔAIC_c_ = –10.7), and sex was not a significant predictor of percent change in overt reaction distance (β = –13.4, CI = –60.7, 33.9, *P* = 0.560). Similarly, among individuals in the AC Group, sound treatment did not affect percent change in overt reaction distance (β = 0.5, CI = –68.0, 69.1, *P* = 0.987).

The results for displacement distance were similar; the AC Group was associated with a significant increase (69.0%) in displacement distance from 13.6m ± 3.4 to 22.7m ± 6.7 (β = 84.4, CI = 47.8, 121.1, *P* < 0.001), compared to a 15.4% decrease from 20.0m ± 10.2 to 14.2m ± 4.6 in displacement distance for bears in the Control Group (Fig 1B), and the model including AC Group had significantly better performance than the null model (ΔAIC_c_ = 14.2, *P* < 0.001). Again, adding sex to the model worsened model fit (ΔAIC_c_ = –1.7), and neither sex (β = –18.9, CI = –53.2, 15.4, *P* = 0.294) nor sound treatment (β = 21.9, CI = –25.5, 69.4, *P* = 0.333) were significant predictors of change in displacement distance. Adjusted R^2^ for the overt reaction distance and displacement distance models were 0.36 and 0.51, respectively, suggesting that displacement distance was the more responsive variable following AC.

We further tested the influence of AC programs on bear wariness by determining bear response to researchers for 314 AC events in which bear response was recorded prior to beginning AC treatment. Prior to conducting the AC event, bears left at a run in 33.3% of instances (*n* = 105), left at a walk in 16.5% of instances (*n* = 52), and climbed a tree 3 times. Undesirable responses to researchers included bears exhibiting stress but not displacing (25.7%, *n* = 82), indifference (19.1%, *n* = 61), or approaching researchers (3.4%, *n* = 11). A Fisher test indicated that individual bear ID was an important predictor of whether a bear fled (*P* = 0.030), supporting the use of a GLMM including Bear ID as a random effect. The predictors in two univariate models met our criteria for inclusion in further modelling; these models suggested that a greater number of AC events in the past 30 days increased the likelihood of a bear departing upon researcher arrival, whereas a whistle being blown decreased this likelihood (Table 1). Among the three possible models generated using these two covariates, a GLMM including both the number of previous AC events in the past 30 days and whether a whistle was blown as fixed effects had the lowest AIC_c_. This model fit the data significantly better than the null model (ΔAIC_c_ = 17.2, *P* < 0.001) and indicated that, for every additional previous AC event, the likelihood that a bear would flee increased by 4.5% (OR = 1.05, β = 0.04, CI = 0.02, 0.06, *P* < 0.001; Fig 1C). The model also suggested that the likelihood that a bear would flee from researchers decreased by 45% when a whistle was blown, but the confidence intervals for this term overlapped zero (OR = 0.55, β = –0.60, CI = –1.4, 0.1, *P* = 0.106). Nakagawa marginal and conditional pseudo R^2^ were 0.101 and 0.121, respectively, indicating that 2.0% of the variation in the data was attributable to bear ID, and the remaining 10.1% was explained by the number of AC events in the previous 30 days and whether a whistle was blown. Including distance to cover, sex, and start distance as additional predictors did not improve model performance.

**Table 1:** Performance metrics for univariate models predicting the likelihood that a bear would leave prior to researchers initiating AC tactics, Whistler, BC, 2006–2008. We compared models to a model including bear ID as a random effect and report the beta coefficient estimate (β) and associated 95% confidence interval (CI), P-value (*P*), odds ratio (OR) and 95% CI, sample size (N), difference in Akaike Information Criterion adjusted for small sample size (ΔAIC_c_) compared to a null model and the *P*-value for a likelihood ratio test comparing with the null model (Null).

Predictor	β (CI)	*P*	OR (CI)	N	ΔAIC_c_	Null
**No. AC (last 30 days)**	0.04 (0.0, 0.1)	< 0.001	1.05 (1.0, 1.1)	314	16.9	< 0.001
**Whistle Blown**	–0.62 (–1.4, 0.1)	0.093	0.54 (0.3, 1.1)	313	0.85	0.089
**Distance to Cover (m)**	–0.01 (0.0, 0.0)	0.363	0.99 (1.0,1.0)	274	–1.21	0.362
**Sex (M)**	0.27 (–0.6, 1.0)	0.467	1.31 (0.6, 2.8)	314	–1.55	0.484
**Start Distance (m)**	0.00 (0.0, 0.0)	0.705	1.00 (1.0,1.0)	56	–2.1	0.704

### Success of individual AC events

**Fig 2:**
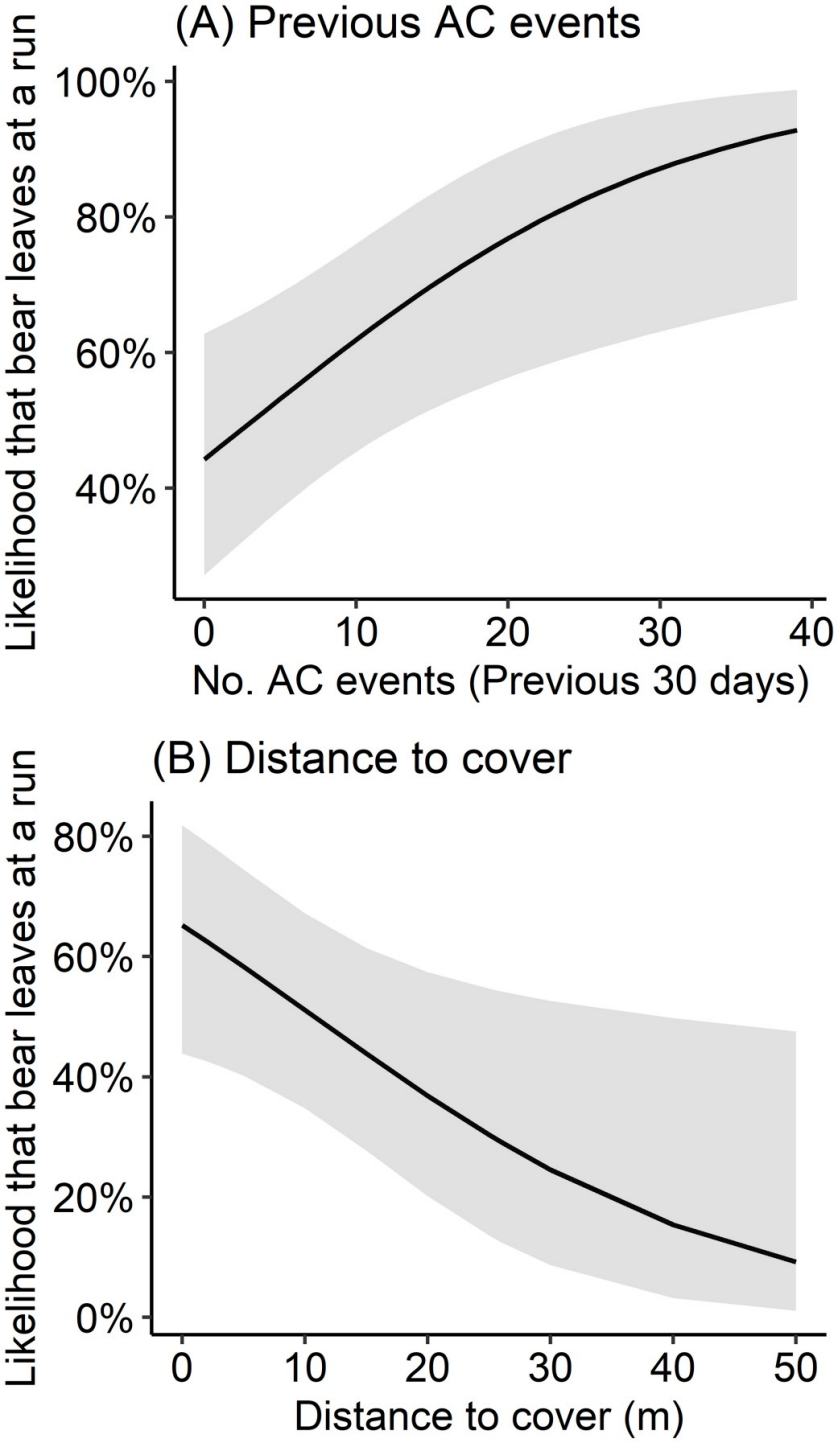
The predicted effects of the number of AC events in the previous 30 days (A) and distance to cover (m; B) on the likelihood that a bear subjected to an AC event will leave at a run, Whistler, British Columbia, 2007–2008.

We considered 115 events in which AC was conducted to assess the predictors of success for individual AC events, which we defined as bears leaving at a run. Bears ran in 68 events (59.1%) and left at a walk or failed to displace in 47 events (40.9%). A Fisher test indicated that bear ID significantly affected event outcome (*P* = 0.005). Among GLMMs that contained a single fixed effect, only the number of AC events in the previous 30 days and distance to cover met our criteria for inclusion (Table 2). Although bears were 4.8 times more likely to flee from projectiles launched from guns compared to those launched from slingshots (OR = 4.77), this effect was associated with a small sample size (*n* = 37) and wide confidence intervals that overlapped zero (β = 1.56, *P* = 0.159, CI = –0.4, 4.5). Model performance was not improved by including the variables of sex, start distance, and whether or not a whistle was blown (Table 2). To create a final model, we used the 103 events for which distance to cover had been recorded and used this term and the number of AC events in the previous 30 days as fixed effects. This model suggested that for each additional AC event in the last 30 days, likelihood of a successful AC event increased by 7% (OR = 1.07, β = 0.07, *P* = 0.008, CI = 0.022, 0.12; Fig 2A), and that every additional m from cover decreased likelihood of success by 6% (OR = 1.07, β = 0.07, *P* = 0.008, CI = 0.022, 0.12; Fig 2B). The model performed better than the more predictive of the two univariate GLMMs (ΔAIC_c_ = 3.08, *P* = 0.022). Nakagawa conditional and marginal pseudo R^2^ values suggested that 24.5% of the variation in the data was explained by the number of AC events in the past 30 days and distance to cover, and the remaining 13.7% was attributable to individual bear ID.

**Table 2:** Summary statistics and performance metrics for univariate models predicting the likelihood that an AC event would succeed (i.e., a bear would leave at a run), Whistler, BC, 2007–2008. All models included bear ID as a random effect and were compared to a null model including only this term. Metrics reported include the beta coefficient estimate (β) and associated 95% confidence interval (CI), *P*-value (*P*), odds ratio (OR) and 95% CI, sample size (N), difference in Akaike Information Criterion adjusted for small sample size (ΔAIC_c_) compared to a null model and the *P*-value for a likelihood ratio test comparing with the null model (Null).

Predictor	β (CI)	*P*	OR (CI)	N	ΔAIC_c_	Null
**No. AC (last 30 days)**	0.09 (0.0, 0.1)	0.001	1.09 (1.0, 1.1)	115	12.5	< 0.001
**Distance to Cover (m)**	–0.07 (–0.1, 0.0)	0.004	0.93 (0.9, 1.0)	103	7.3	0.002
**Projectile Type (Gun)**	1.56 (–0.4, 4.5)	0.159	4.77 (0.5, 42.0)	37	–0.01	0.124
**Start distance**	–0.01 (0.0, 0.0)	0.182	0.99 (1.0, 1.0)	66	–0.22	0.160
**Sex (M)**	0.82 (–0.6, 2.3)	0.241	2.28 (0.6, 9.0)	115	–0.77	0.248
**Whistle blown**	–1.22 (–4.3, 1.1)	0.328	0.29 (0.0, 3.4)	89	–1.07	0.301

### Spatial and temporal patterns

**Fig 3:**
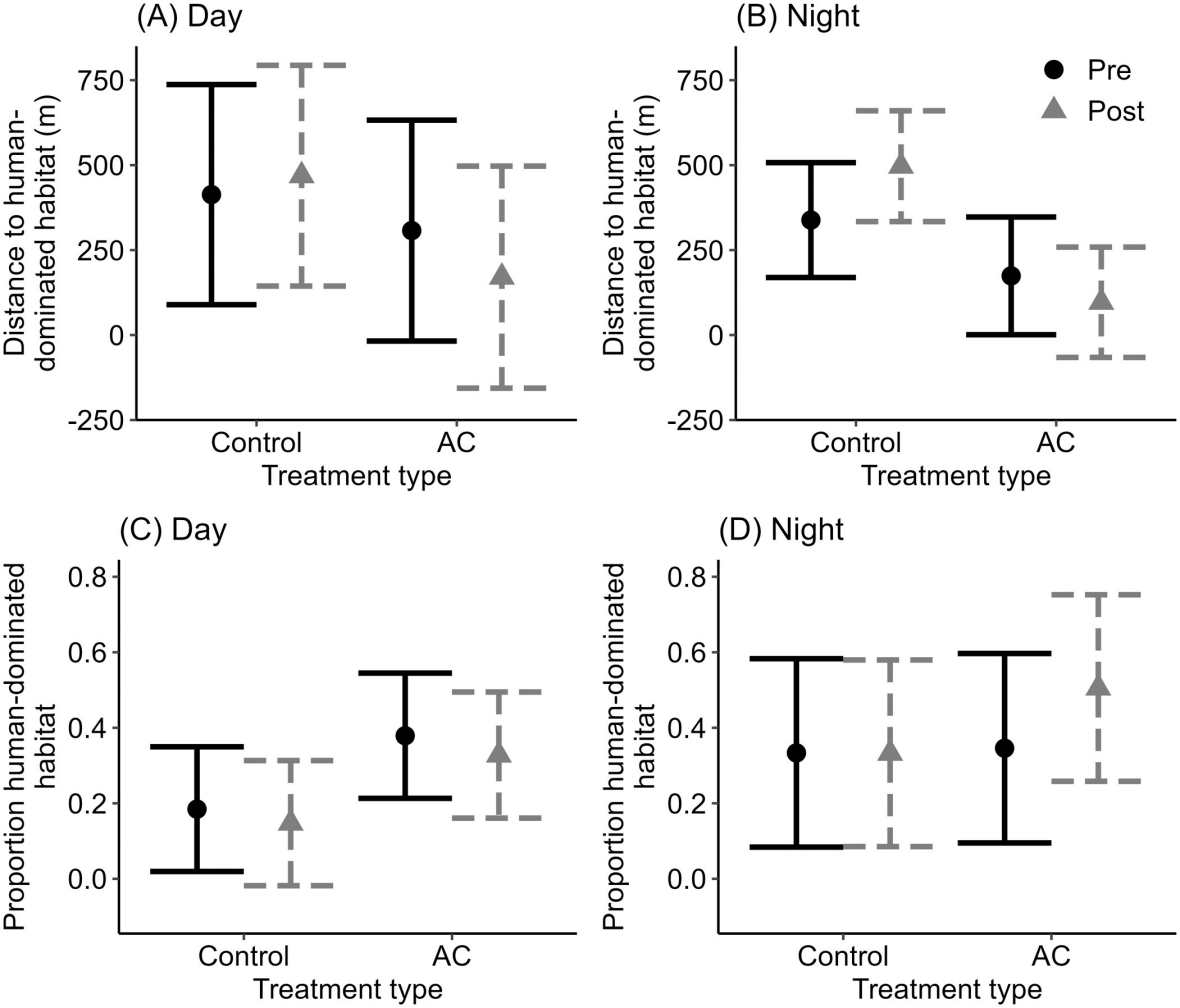
Effects of AC programs on use of human-dominated habitat by black bears during the day and at night, Whistler, BC, 2007–2008. The top panels show the distance to human-dominated habitat during the day (A) and at night (B) among bears in the AC Group and the Control Group before and after AC programs. The lower panels show the proportion of human-dominated habitat within 100-m of bear location during the day (C) and at night (D) among the same groups. Vertical bars represent 95% confidence intervals.

Of the nine bears fitted with GPS collars, one was killed by a vehicle early in the monitoring period, leaving four GPS-collared bears in each of the AC and Control Group for study of spatial and temporal patterns. We used 859 GPS locations for this analysis, which included 610 daytime and 249 nighttime locations that were collected between May and November in 2007–2008. Bears in the AC Group were 50.5% closer to human-dominated habitat during the day (interaction term β = –192.6, *P* = 0.031; Fig 3A, Table 3) and 38.9% closer to human-dominated habitat at night (interaction term β = –236.3, *P* = 0.067; Fig 3B, Table 3). By contrast, bears in the Control Group were on average 12.0% further from human-dominated habitat during the day (Fig 3A, Table 3) and 54.1% further from human-dominated habitat at night (Fig 3B, Table 3). Both models lowered AIC_c_ compared to the null, but only the model for nighttime locations fit the data better than the null (*P* = 0.012). Compared to the pre-treatment period, the proportion of human-dominated habitat within 100-m of AC-treated bears decreased by 9.3% during the day (interaction term β = –0.014, *P* = 0.786; Fig 3C, Table 3) and increased by 52.7% during the night (interaction term β = 0.160, *P* = 0.078; Fig 3D, Table 3). Among bears in the Control Group, the proportion of human-dominated habitat within 100-m decreased following designated treatment periods during both the day (20.3%; Fig 3C; Table 3) and the night (8.5%; Fig 3D, Table 3). These were also weak models and performed worse than the null models for both daytime and nighttime locations.

**Table 3:** Summary statistics and performance metrics for models predicting distance to human-dominated habitat (distance) and proportion of human-dominated habitat within 100 m (proportion) prior to and following AC programs during the day and at night, Whistler, BC, 2007–2008. All models included bear ID as a random effect and were compared to a null model including only this term. Metrics reported include the beta coefficient estimate (β) and associated 95% confidence interval (CI), *P*-value (*P*), difference in Akaike Information Criterion adjusted for small sample size (ΔAIC_c_) compared to a null model and the *P*-value for a likelihood ratio test comparing with the null model (Null).

	AC Group		Post AC Program		AC Group * Post AC Program		Comparison to Null	
Outcome	β (CI)	*P*	β (CI)	*P*	β (CI)	*P*	ΔAIC_c_	*P*
**Distance (Day)**	–105.9 (–553.7, 342.3)	0.665	55.7 (–64.7, 175.9)	0.365	–192.6 (–367.4, –17.9)	0.031	–32.9	0.101
**Distance (Night)**	–164.2 (–397.9, 67.0)	0.208	158.6 (–13.5, 337.0)	0.078	–236.3 (–488.8, 11.6)	0.067	–36.5	0.012
**Proportion (Day)**	0.194 (–0.03, 0.42)	0.150	–0.037 (–0.11, 0.03)	0.301	–0.014 (–0.12, 0.09)	0.786	12.7	0.122
**Proportion (Night)**	0.012 (–0.33, 0.36)	0.947	–0.001 (–0.13, 0.12)	0.988	0.160 (–0.02, 0.34)	0.078	8.9	0.090

## Discussion

Increasing human-black bear conflict and growing public opposition to lethal management techniques emphasize the importance of developing and testing effective non-lethal tools for addressing conflict between people and bears. In this study, we tested several aspects of AC as a method for reducing human-black bear conflict in the Resort Municipality of Whistler, which has extensive integration of residential, commercial, and natural areas. We found that brief (i.e., 3–5 day) AC programs were effective at increasing short-term wariness in conflict bears, and that individual AC events were most successful at causing bears to run from conditioning when they had been subjected to AC previously and when they were close to cover. AC did not reduce bear use of human-dominated habitat during the day or at night. Our results show that AC may deter bears from conflict situations in the short term but suggest that broader conflict reduction will require other proactive management tools, especially those that prevent bears from becoming food-conditioned.

Among the individuals treated in this study, AC consistently increased bear wariness towards humans as demonstrated by increased overt reaction distance and displacement distance following AC programs, and the positive association between the number of previous AC events and likelihood that a bear would flee prior to researchers beginning AC tactics. By contrast, bears in the Control Group became increasingly habituated to humans, as demonstrated by decreasing overt reaction distance and displacement distance. This result is consistent with other study findings suggesting that AC increases wariness in black bears [33, 48, 50, 51] and other carnivores, such as coyotes (*Canis latrans*) [69, 70], African lions (*Panthera leo*) [71], and wolves (*Canis lupus*) [72]. The relative effectiveness of these AC programs for increasing wariness could relate to several aspects of program implementation. Because we subjected bears to aversive stimuli as they engaged in problematic behaviour [48, 50], we increased the likelihood that bears associated the conditioning stimulus (conflict behaviour) with the unconditioned stimulus (pain/ stress) [38, 52]. This principle of immediacy in aversive conditioning [54] is not achieved when aversive conditioning occurs upon release of a captured bear, sometimes hours later and kilometres distant from the capture location where conflict occurred [32]. Repetition of treatments allowed bears to generalize among experiences instead of associating the painful stimulus with a single location or human individual, which has been identified as important to AC programs targeting bold coyotes [69], lions associated with livestock depredation [71], and wolves [72]. Unfortunately, both immediacy in aversive conditioning and repetition are costly to implement and impractical in some circumstances, particularly in jurisdictions like Whistler where a single conservation officer may need to respond to calls over several hundred square kilometers. Ongoing availability of anthropogenic attractants reduced the efficacy of some AC programs [38, 52], so our program may have been supported by pre-existing and long-term efforts to control anthropogenic attractants in Whistler. In concert with other studies, our results suggest that AC may be most effective at reducing conflict as an early intervention, prior to bears becoming strongly food-conditioned, which is also more cost effective and acceptable to the public [30, 48, 50].

The success of individual AC events was predicted by individual ID, more previous AC events, and proximity to cover. AC success varies among individuals in black bears [49, 50], grizzly bears [52], elk (*Cervus canadensis*) [66], and lions [71]. The positive influence of previous AC events suggests bears become increasingly sensitized to AC; repetition may allow bears to generalize learning to conflict behaviour rather than specific sites, situations, or human individuals [48, 52]. Proximity to cover may increase likelihood of bear departure because a bear with nearby cover may flee into secure habitat, whereas a bear without nearby cover may be more likely to stand its ground [73, 74]. Alternately, bolder individuals could be both more likely to use areas far from cover and be less likely to flee from AC [52, 66]. Other predictors associated with wide confidence intervals revealed interesting patterns that could be explored in future studies. Weak evidence suggested that projectiles from firearms more effectively deterred bears than those fired from slingshots, which is consistent with previous findings in bears [48]. Given the limitations of firearms in human-dominated areas and the necessity that they be operated by trained personnel [38, 48], further exploration of slingshots as a bear deterrent is warranted, though studies should include multiple metrics of success, as bears may immediately respond to slingshots and firearms the same way, but stay away longer after treatment with firearms [48]. Weak evidence suggested males were more sensitive to AC, which supports the idea that AC success depends on age-sex class [27, 50, 71] and that males are often more wary than females, who may take more risks to support offspring [75, 76]. Consistent with another study [52], sound treatment did not increase any of our metrics of success. Unexpectedly, whistle use decreased the likelihood that bears would flee, perhaps suggesting that bears were curious about the sound, as has been reported with other novel sounds, such as bear bells [77, 78]. Because predictors of successful AC events were rarely indicated in the literature, we suggest that this type of information be more frequently reported so that the contexts where AC is most successful are apparent.

Bears did not change their use of human-dominated habitat following AC at night or during the day, which is consistent with studies of coyotes [70] and lions [71]. Some other studies corroborate our result that bears continued to use or returned to their previous habitat following AC [33, 34, 49, 51], but other studies showed that black bears reduced daytime use of human-dominated areas following AC [48, 50]. The metrics we used to represent potential for human-black bear conflict (i.e., distance to human-dominated habitat and proportion of human-dominated habitat within 100 m) may not be good measures of actual conflict, so they have limited utility in assessing program success. For example, a bear that responds to AC by avoiding a public trail during the day, but sleeps nearby in dense cover would demonstrate no behavioural change in our metrics, although the likelihood of conflict is presumably quite reduced. Unfortunately, our measures could not assess such subtle effects of AC on habitat use. Because increased wariness to people is a successful outcome of behavioural interventions for bears [52], effective metrics for measuring it should be developed along with other metrics of success for AC programs. Metrics of habitat use are likely to be most powerful if they interact with more direct metrics of human-bear conflict, such as conflict reports submitted by the community [69], or quantification of anthropogenic food in bear scat [79].

This study had several limitations, the first of which is that some data were missing, which limited our initial modelling approach to univariate tests and reduced analytical power. Operational programs employing AC will typically have this limitation because many individuals collect data, seasonal employment may limit record keeping, and it is challenging to collect data while conducting AC as part of an active management program. Combined with the small sample sizes characteristic of these programs, this phenomenon may partially explain why many AC studies are published only in the gray literature or remain unreported altogether, despite the ubiquity of AC as part of management plans for bears [27] and other carnivores [69]. Future studies should attempt to quantify individual boldness [49], and include bear age [48] and health [52] as predictors. We also observed bears and conducted AC at different times of year; seasonal changes in animal behaviour may have affected results, and future studies could seek to conduct data collection at specific times of year or account for season in modelling approaches. Although all bears in this study were associated with conflict, we were unable to assess food-conditioning, which is an important predictor of AC program success [48]. Some individuals may have been deeply food-conditioned and dependent on anthropogenic resources, while others may have been merely habituated. All of these differences among bears may have contributed to the large effects of individual ID in our analyses.

## Conclusion

Overall, this study demonstrates the utility of AC for increasing short-term wariness in conflict black bears and reinforces the need for AC programs to feature repeated treatments that occur during or shortly after bears engage in conflict behaviour. For AC to meaningfully reduce human-bear conflict, it should be part of a suite of management tools that emphasize proactive measures, such as public education, attractant management, and prevention of food-conditioning [28]. Standardization of AC protocols and increased data sharing among management agencies could alleviate problems associated with small sample sizes, allowing critical information about the effectiveness of AC to be integrated and shared outside of the gray literature. We suggest that management agencies strive to record AC data consistently, which may be facilitated with mobile phone apps [80, 81]. Together, these actions could maximize the efficacy and humaneness of AC as a non-lethal management tool for addressing human-bear conflict.

## Supporting information

S1 Table A summary of the sex, capture year, collar type, and treatment information for all bears included in the study, Whistler, British Columbia, 2006–2008. (DOCX)
