# Supplementary material for: Aversive conditioning increases short-term wariness but does not change habitat use in black bears associated with conflict
Source: PLoS One. 2024 Jan 2;19(1):e0295989. doi: 10.1371/journal.pone.0295989 (PMC10760891; doi:10.1371/journal.pone.0295989)
Supplement: S1 Table — (DOCX) [file pone.0295989.s001.docx]

**Table S1. A summary of the sex, capture year, collar type, and treatment information for all bears included in the study, Whistler, British Columbia, 2006 – 2008.**

| Bear ID | | Sex | Capture | Collar | Treatment Group | Sound Group |
| --- | --- | --- | --- | --- | --- | --- |
| F01 | F | | 2005 | Radio | Treatment | Sound |
| F02 | F | | 2005 | Radio | Treatment | No Sound |
| F04 | F | | 2006 | GPS | Treatment | No Sound |
| F05 | F | | 2006 | GPS | Control | N/A |
| F07 | F | | 2006 | Radio | Treatment | No Sound |
| F10 | F | | 2007 | GPS | Initially control, later treatment | Sound |
| F11 | F | | 2007 | Radio | Treatment | No Sound |
| F12 | F | | 2007 | GPS | Control | N/A |
| F13 | F | | 2007 | GPS | Treatment | No Sound |
| F18 | F | | DNR | Radio | Control | N/A |
| M01 | M | | 2005 | Radio | Treatment | No Sound |
| M05 | M | | 2005 | Radio | Control | N/A |
| M14 | M | | 2006 | Radio | Treatment | No Sound |
| M17 | M | | 2006 | GPS | Treatment | Sound |
| M19 | M | | 2006 | Radio | Treatment | No Sound |
| M20 | M | | 2007 | Radio | Treatment | No Sound |
| M21 | M | | 2007 | GPS | Control | N/A |
| M23 | M | | 2007 | GPS | Treatment | Sound |
| M25 | M | | 2007 | Radio | Treatment | No Sound |
| M27 | M | | 2007 | Radio | Treatment | No Sound |
| M28 | M | | 2007 | GPS | Treatment | Sound |
| M29 | M | | 2007 | Radio | Treatment | No Sound |
| M30 | M | | 2007 | Radio | Treatment | No Sound |
| M31 | M | | 2007 | Radio | Treatment | No Sound |
| M32 | M | | 2007 | Radio | Treatment | No Sound |
| M33 | M | | DNR | Radio | Treatment | No Sound |
| M38 | M | | DNR | Radio | Treatment | Sound |
| M39 | M | | DNR | Radio | Initially control, later treatment | Sound |
